# REST overexpression in mice causes deficits in spontaneous locomotion

**DOI:** 10.1038/s41598-018-29441-3

**Published:** 2018-08-14

**Authors:** Li Lu, Anantha Marisetty, Bin Liu, Mohamed Mostafa Kamal, Joy Gumin, Bethany Veo, YouQing Cai, Dina Hamada Kassem, Connie Weng, Mark E. Maynard, Kimberly N. Hood, Gregory N. Fuller, Zhizhong Z. Pan, Matthew D. Cykowski, Pramod K. Dash, Sadhan Majumder

**Affiliations:** 1 Departments of Genetics, The University of Texas MD Anderson Cancer Center, Houston, TX 77030 USA; 2 Department of Neurosurgery, The University of Texas MD Anderson Cancer Center, Houston, TX 77030 USA; 3 Department of Pain Medicine, The University of Texas MD Anderson Cancer Center, Houston, TX 77030 USA; 4 Department of Neurobiology and Anatomy, The University of Texas McGovern Medical School, Houston, TX 77030 USA; 5 Department of Pathology, The University of Texas MD Anderson Cancer Center, Houston, TX 77030 USA; 6 Department of Pathology and Genomic Medicine, Houston Methodist Hospital, Houston, TX 77030 USA; 7 Department of Neuro-oncology, The University of Texas MD Anderson Cancer Center, Houston, TX 77030 USA; 8 Present Address: Baylor College of Medicine, Houston, TX 77030 USA; 9 Present Address: Department of Neurosurgery, MD Anderson Cancer Center, Houston, TX 77030 USA; 10 Present Address: Department of Epigenetics and Molecular Carcinogenesis, MD Anderson Cancer Center, Houston, TX 77030 USA; 11 Present Address: Department of Biochemistry, Faculty of Pharmacy, Ain Shams University, Cairo, Egypt; 12 Present Address: Department of Pediatrics/Hematology and Oncology, University of Colorado Denver Anschutz Medical Campus, Aurora, CO 80045 USA

**Keywords:** Molecular neuroscience, Neuroscience, Genetics

## Abstract

Overexpression of REST has been implicated in brain tumors, ischemic insults, epilepsy, and movement disorders such as Huntington’s disease. However, owing to the lack of a conditional REST overexpression animal model, the mechanism of action of REST overexpression in these disorders has not been established *in vivo*. We created a REST overexpression mouse model using the human REST (*hREST*) gene. Our results using these mice confirm that *hREST* expression parallels endogenous REST expression in embryonic mouse brains. Further analyses indicate that REST represses the dopamine receptor 2 (*Drd2*) gene, which encodes a critical nigrostriatal receptor involved in regulating movement, *in vivo*. Overexpression of REST using *Drd2-Cre* in adult mice results in increased REST and decreased DRD2 expression in the striatum, a major site of DRD2 expression, and phenocopies the spontaneous locomotion deficits seen upon global DRD2 deletion or specific DRD2 deletion from indirect-pathway medium spiny neurons. Thus, our studies using this mouse model not only reveal a new function of REST in regulating spontaneous locomotion but also suggest that REST overexpression in DRD2-expressing cells results in spontaneous locomotion deficits.

## Introduction

The chromatin modifier RE1 silencing transcriptional factor (REST) was first discovered to be a repressor of a few neuronal differentiation genes^1,2^. Since then, REST has been found to potentially regulate thousands of genes impacting both normal development and the diseased state^3–10^. The REST gene is conserved in humans, mice and other vertebrates (https://www.ncbi.nlm.nih.gov/homologene/4099). During mouse embryonic neurogenesis, REST is expressed in neural stem cells (NSCs), and its expression generally diminishes as the NSCs differentiate into mature neurons^3,11–15^. A recently developed full-length *Rest* knock-out mouse model has confirmed REST’s role as a repressor of neurogenesis during mouse embryonic development^16^.

Although the role of REST in mouse embryonic neurogenesis has been well studied, its roles in adult neurons are only now being investigated^13,16–18^. REST is known to be expressed/re-expressed in some mature neurons^11,15^. Transient overexpression of REST during mouse neurogenesis blocked migration and neuronal differentiation^13^. Overexpression (OE) of REST has also been implicated in different types of brain tumors^19–24^, in ischemic insults^25^, in epilepsy^26^, and in movement disorders such as Huntington disease (HD) and Parkinson disease (PD)^11,27^. HD, caused by the mutant huntingtin (*mHTT*) gene, results from an expansion of the CAG trinucleotide repeat of differing length in the *HTT* gene^26,28,29^. Building on studies using the R6/2 HD mouse model and HD patient specimens, it has been postulated that the mHTT protein produces one of its major effects through aberrant REST OE in the neuronal nucleus, presumably through the release of REST from its cytoplasmic HTT-REST complex leading to the accumulation of REST in the nucleus where it represses its target genes^30–32^. Independently, the huntingtin interacting protein 1 protein interactor (HIPPI) has been found to directly activate *REST* gene transcription in an HD model^33^. In addition, decreased levels of miR-9, which targets REST, have also been found in HD patient cortices^34^. These observations suggest multiples mechanism of REST OE in HD. Indeed, REST has been postulated to be a novel therapeutic target in cellular models of HD^35,36^. Although HD has a defined genetic origin, the molecular and cellular mechanisms underlying the disease remain unclear and complex^37^. Overexpression of REST has also been implicated in PD^11^ although the connection between REST and PD is less clear than the connection between REST and HD. An unbiased genome-wide transcriptome analysis of an animal model of PD suggests that REST forms a robust transcriptomic hub in PD resulting in lowered expression of tyrosine hydroxylase (TH) and brain-derived neurotrophic factor (BDNF), two PD markers and direct targets of REST^38^. However, a study using a rat model of PD found concomitant lowered expression of BDNF and overexpression of miR-132, which targets REST^39^. Thus, the role of REST in PD remains controversial.

A roadblock to eliminating this knowledge gap is the lack of a conditional REST overexpression mouse model^15^. Constitutive REST overexpression in mice is embryonically lethal^40^ and, therefore, cannot be used to study these disorders. We have now generated a conditional REST overexpression knock-in mouse model using the human REST (*hREST*) gene. Results using these mice support the expected role of REST as an embryonic neurogenesis repressor. Further, our results suggest that REST represses the *DRD2* gene, and when expressed in DRD2-expressing cells, regulates spontaneous locomotion.

## Results

### Genetically engineered *hRESTloxP/loxP (LSL-hREST)* mouse model

**Figure 1:**
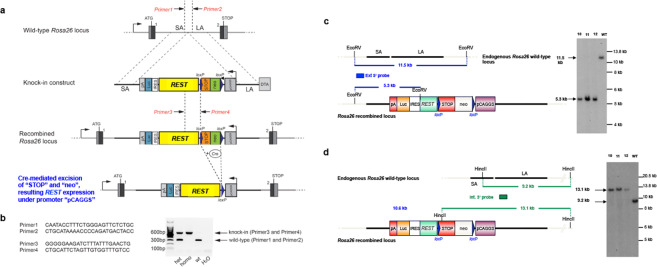
Generation and characterization of LSL-hREST mice. (**a**) Detailed strategy for the creation of a knock-in conditional Rest overexpression *LSL-hREST* mouse model (*loxP-STOP-LoxP-hREST-IRES-Luc* construct inserted at the Rosa26 locus and expressing through the CAGGS promoter). Thus, in the absence of the Cre recombinase, transcription from the CAGGS promoter will terminate at the STOP signal. In contrast, in the presence of Cre, the STOP signal will be excised and the REST gene transcription will take place. (**b**) Genotyping of the transgenic mice using polymerase chain reaction assays with probes described in the figure. (**c**,**d**) Southern blot analysis for homologous recombination in embryonic stem cells. (**c**) 5′ homologous recombination. Schematic representation of the wild-type (top) and recombinant (bottom) Rosa26 alleles with the relevant restriction sites and probe for Southern blot analysis is shown. The genomic DNA of the tested embryonic stem cell clones was compared with wild-type C57BL/6 genomic DNA (WT). The digested DNA was blotted on nylon membranes and hybridized with the 5′ probe detecting the BamHI fragment to screen for 5′ homologous recombination events. M: 1 kb DNA-Ladder (Fermentas). (**d**) 3′ homologous recombination. Schematic representation of the wild-type and recombinant Rosa26 alleles with the relevant restriction sites and probe for Southern blot analysis. The genomic DNA of the tested embryonic stem cell clones was compared with wild-type C57BL/6 genomic DNA (WT). The digested DNA was blotted on nylon membranes and hybridized with the 3′ probe detecting the HincII fragment to screen for 3′ homologous recombination events. M: 1 kb DNA-Ladder (Fermentas). Figures represent at least three different experiments. Blots shown in B, C, and D are from the same gel without cropping.

**Figure 2:**
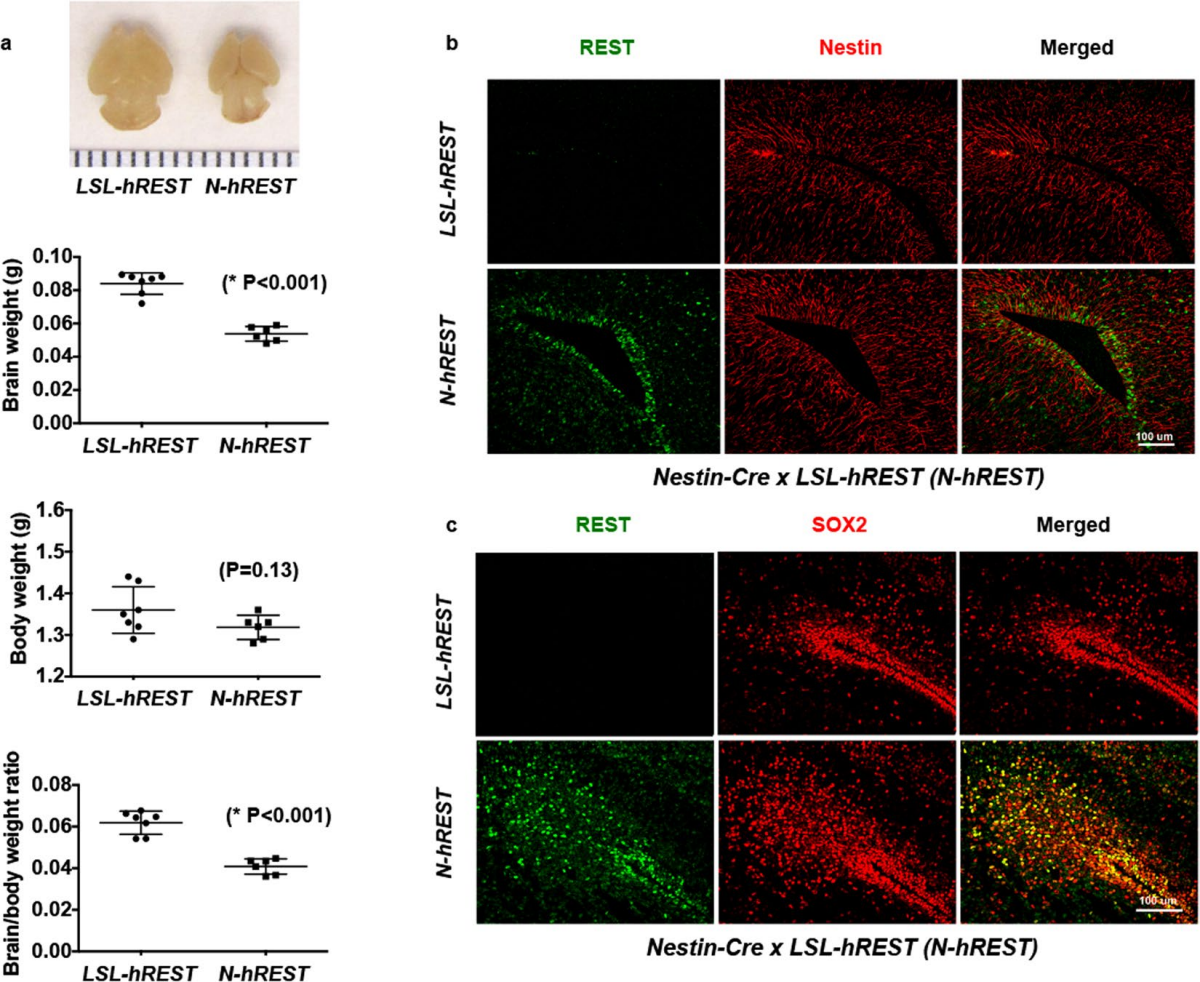
Characterization of *N-hREST* mice. (**a**) *Nestin-Cre x LSL-hREST* (*N-hREST*) heterozygous pups were born dead and had smaller brains than the *LSL-hREST* control pups. Body, brain weight, and brain/body weight of E18.5 embryos are shown. Experiments were performed at least in triplicate. P values are shown in the figure. (**b**,**c**) REST expression in *N-hREST* mouse brains correlates with stemness in embryonic neural stem cells. Immunofluorescence analysis of E18.5 *N-hREST* and *LSL-hREST* control littermate mouse brains with antibodies against REST (using an antibody that preferentially recognizes hREST over mouse REST) and Nestin (**b**) and REST and SOX2 (**c**). Figures represent at least three different images. Scale bar for B and C = 100 μ.

To gain insight into the process of REST overexpression, we generated a conditional mouse model (*hRESTloxP/loxP* = *LSL-hREST*) in which a *loxP-STOP-LoxP-human REST (hREST)-IRES-Luc* construct expressing the exogenous *hREST* gene through the CAGGS promoter was inserted at the Rosa26 locus (Fig. 1a). The transgenic mice were genotyped using PCR (Fig. 1b) and the homologous recombination was confirmed using Southern blotting analyses (Fig. 1c,d). To express the exogenous *hREST* in neural stem cells (NSCs), we crossed *LSL-hREST* mice with *Nestin-Cre* mice. The homozygous *N-hRESTov/ov* pups had embryonic lethality. The heterozygous pups (*N-hRESTov*/+ = *N-hREST*) were dead upon birth and had 30% smaller brains (Fig. 2a) than the control *LSL-hREST* littermates. Double immunofluorescence analysis of REST and Nestin protein expression in the brains of E18.5 *N-hREST* embryos indicated that exogenous hREST was expressed in many, but not all, Nestin-positive cells, and *hREST* expression was more prevalent in cells located around the ventricles (Fig. 2b).

Because endogenous REST expression is mostly limited to SOX2-positive cells in the embryonic brain^16^, we wanted to determine whether the exogenous *hREST* expression in E18.5 *N-hREST* embryo brains was also seen in SOX2-positive cells. Double immunofluorescence analysis of hREST (using an antibody that preferentially recognizes hREST over mouse REST) and SOX2, a neural stem cell regulator^41^, suggested that most SOX2-expressing cells, particularly cells surrounding the ventricles, co-expressed hREST (Fig. 2c). These results were very similar to what was observed in another publication^16^ and suggest that the expression of exogenous hREST parallels that of endogenous mouse REST.

### REST suppresses Drd2 transcription in N-hREST mice

**Figure 3:**
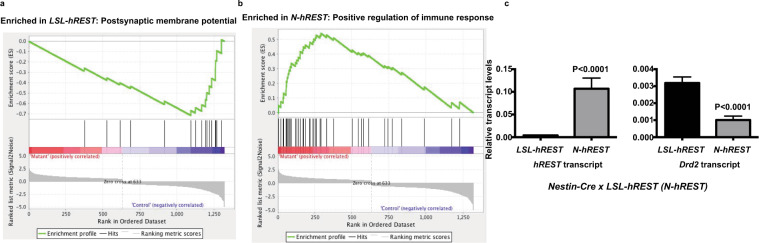
REST suppresses Drd2 expression in N-hREST mice. (**a**,**b**) REST overexpression causes a global shift in genome-wide transcriptome profiles of E18.5 *LSL-hREST* control littermate (**a**) versus *N-hREST* (**b**) mouse brains. RNA-Seq analyses were performed in triplicate. (**c**) Inverse expression patterns of *hREST* and *Drd2* transcripts. Quantitative real-time polymerase chain reaction analyses for *hREST* and *Drd2* transcripts of E18.5 *N-hREST* and control littermate mouse brains. Experiments in c were performed in triplicate. P values are shown in the figure.

To define the REST-regulated transcriptome, we performed RNA-Seq analyses of the brains of E18.5 *N-hREST* mice and their *LSL-hREST* control littermates. To examine the functions of genes in the transcriptome profiles, we utilized GSEA using the normalized significantly different gene expression values for the *LSL-hREST* control littermate (Supplementary Table S1) and *N-hREST* (Supplementary Table S2) mouse brains. The gene sets in this analysis were from the Gene Ontology biological process category. While most of the gene sets enriched in the control littermate brains were neuron-related, such as the gene set associated with postsynaptic membrane potential (Fig. 3a), those enriched in the *N-hREST* mouse brains belonged to immune-related gene sets such as the gene set associated with positive regulation of immune response (Fig. 3b). These results suggested that there was a global shift in transcriptional regulatory networks in the *N-hREST* mouse brain.

The RNA-Seq analysis suggested many gene transcripts that were downregulated owing to REST overexpression, including known REST target genes such as *Neurod1* and *Syn* (Gao *et al*., 2011), *Syp* and *Snap25* (Nechiporuk *et al*., 2015), and *Syt4, Chga*, and *Scg2* (Watanabe *et al*., 2004). The analysis also suggested *Drd2*, which encodes a critical nigrostriatal receptor, was found to be a REST target in a previous genome-wide chromatin REST-binding screen^42^. We were interested in REST-DRD2 regulation because many studies have suggested a link between DRD2 and various motor disorders^43–49^.

To validate our RNA-Seq bioinformatic finding that REST regulates DRD2 in *N-hREST* mice, we performed transcript analyses of the brains from E18.5 *N-hREST* mice, which served as an *in vivo* REST gain-of-function system, and their *LSL-hREST* control littermates. As expected, the *N-hREST* mouse brains had higher expression of *hREST* and lower expression of *Drd2* transcripts compared with their wild-type littermates (Fig. 3c), suggesting that REST regulates DRD2 expression.

**Figure 4:**
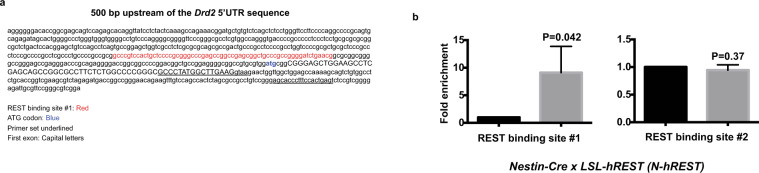
REST binds to a specific REST binding site present in the *Drd2* gene chromatin. (**a**) Potential REST binding sites within 500 bp upstream of the *Drd2* gene chromatin. REST binding sites are shown red. Exon 1 is shown in capital letters. (**b**) Quantitative chromatin immunoprecipitation analyses of neurospheres obtained from E12.5 wild-type mouse brains using REST and IgG control antibodies followed by polymerase chain reaction using primers corresponding to a predicted REST binding site and a non-specific site. Experiments in b were performed in triplicate. P values are shown in the figure.

We wanted to examine whether REST directly binds to the *Drd2* gene chromatin. We performed quantitative chromatin immunoprecipitation assays (qChIP) analyses of neurospheres generated from wild-type mouse brains. Bioinformatic analyses of the *Drd2* promoter elements 500 bp upstream of the 5′ untranslated region (5′-UTR) found a few potential REST binding sites, including the one closest to the 5′ UTR (site #1: 80 bp upstream of ATG; Fig. 4a). Site #1 contained three overlapping REST binding sites. We searched 7 kb downstream of the 5′ UTR sequence and picked another potential binding site (site #2: 6.1 kb downstream of ATG) and performed quantitative ChIP assays using either anti-REST or IgG control antibodies. REST bound to site #1 but not to site #2, which suggests specific REST binding on the *Drd2* chromatin at site #1 (Fig. 4b).

### REST inhibits Drd2 expression in D-hREST mice

**Figure 5:**
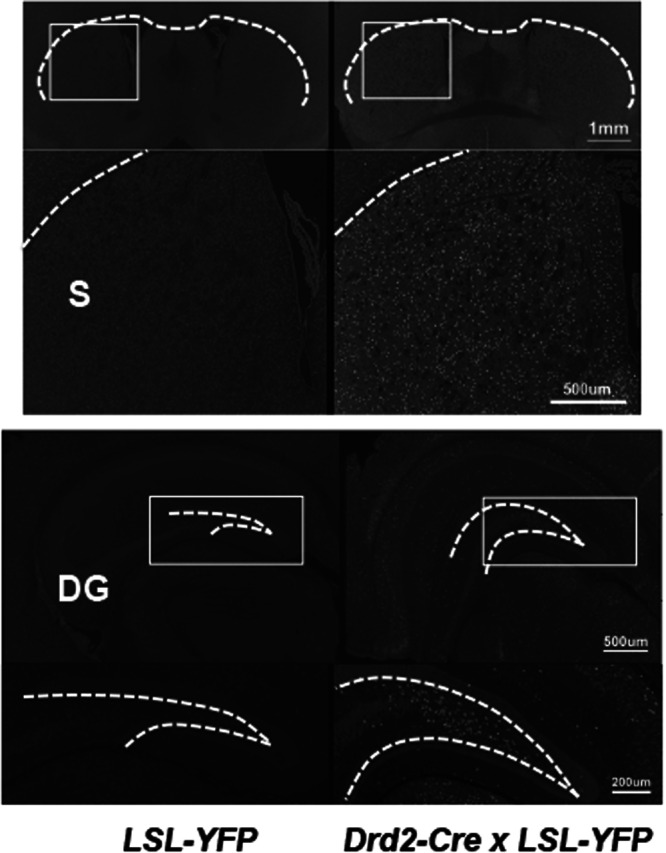
Specificity of Cre expression in Drd2-Cre mice. Yellow fluorescent protein (YFP) immunofluorescence analyses of the mouse brains obtained from *Drd2-Cre x LSL-YFP* reporter mice. Low magnification for striatum (scale bar = 1 mm) and dentate gyrus (scale bar = 500 µm) as well as magnified views of the marked areas (white boxes) for striatum (scale bar = 500 µm) and dentate gyrus (scale bar = 200 µm) are shown. YFP (Cre recombinase) was mostly and robustly expressed in the striatum (S). Limited Cre expression was also seen in other areas of the brain such as the dentate gyrus (DG). Figures represent at least three different images.

To determine the impact of REST expression in DRD2-positive cells, we obtained *Drd2-Cre* mice. First, to validate the specificity of the Cre expression in these mice, we crossed the *Drd2-Cre* mice with *LSL-YFP* reporter mice. We used the reporter *LSL-YFP* mice in this cross to allow Cre-inducible expression of YFP in DRD2-expressing target cells. Analysis of YFP immunofluorescence in the resulting *D-YFP* mouse brain sections suggested that the Cre recombinase was robustly expressed in the striatum (Fig. 5), as expected (www.gensat.org/cre). Striatum is a major brain site affected in movement disorder patients^44^.

**Figure 6:**
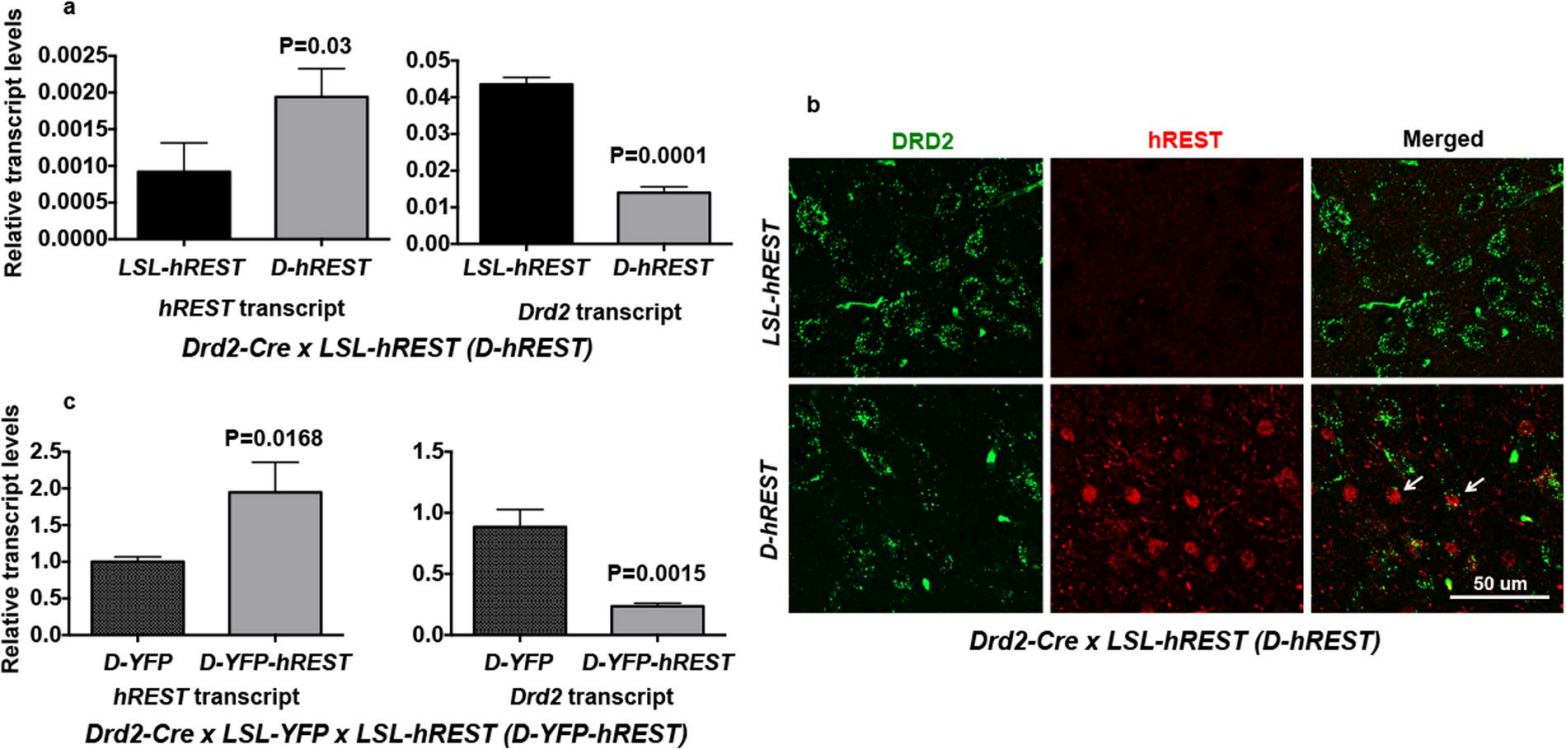
REST inhibits DRD2 expression in adult D-hREST mice. (**a**) Inverse expression patterns of *hREST* and *Drd2* transcripts. Quantitative real-time polymerase chain reaction analyses for *hREST* and *Drd2* transcripts of 10- to 12-week-old *D-hREST* and control littermate mouse brains. Experiments were performed in triplicate. (**b**) Inverse expression patterns of hREST and DRD2 proteins. Immunofluorescence analysis of the brain striatum of *D-hREST* and control littermate mice using antibodies against REST and DRD2. Figures represent at least three different images. Scale bar = 50 µm. Arrows show cells expressing both hREST and DRD2 (albeit at a low level), suggesting that the inverse REST-DRD2 expression pattern is not due to the lack of REST expression in DRD2-expressing cells. (**c**) Inverse expression pattern of *hREST* and *Drd2* transcripts in sorted Cre-expressing target cells. Quantitative real-time polymerase chain reaction analyses for *hREST* and *DRD2* transcripts of YFP-sorted cells from adult *D-hRESTxLSL-YFP* and control littermate mouse brains. Experiments were performed in triplicate. P values are shown in the figure.

We then crossed the *LSL-hREST* mice with the *Drd2-Cre* mice. We obtained heterozygous *D-hREST*ov/+ (*D-hREST)* mice to confirm that REST regulates DRD2 in these mice. We performed transcript analyses of the brain striata of 10- to 12-week-old *D-hREST* mice and their *LSL-hREST* control littermates. As expected, the *D-hREST* mouse striata had higher expression of *hREST* and lower expression of *Drd2* transcripts than did the striata of their *LSL-hREST* control littermates (Fig. 6a). Double immunofluorescent analyses of the striata showed moderate levels of REST protein overexpression in the *D-hREST* mice (Fig. 6b). While some REST-expressing cells showed undetectable expression of DRD2, other REST-expressing cells showed low levels of DRD2 expression (Fig. 6b, arrows). The latter observation suggested that the inverse REST-DRD2 expression pattern is not due to the lack of REST expression in DRD2-expressing cells. Overall, REST expression levels were consistently associated with lower levels of DRD2 expression. Taken together, these results suggested that REST suppresses DRD2 expression *in vivo*.

Because the brain striata contained many cell types in addition to the DRD2-expressing cells, we generated *Drd2-Cre x LSL-YFP x LSL-hREST (D-YFP-hREST)* mice. We used fluorescence sorting to identify striatum cells with YFP expression and performed qRT-PCR analyses on those cells. As shown, purified YFP+ cells from *D-YFP-hREST* mice had higher expression of *hREST* and lower expression of *Drd2* transcripts than did the cells from the control mice (Fig. 6c). Taken together, these results suggest that REST represses *Drd2*.

### REST regulates spontaneous locomotion in *D-hREST* mice and phenocopies DRD2 loss

**Figure 7:**
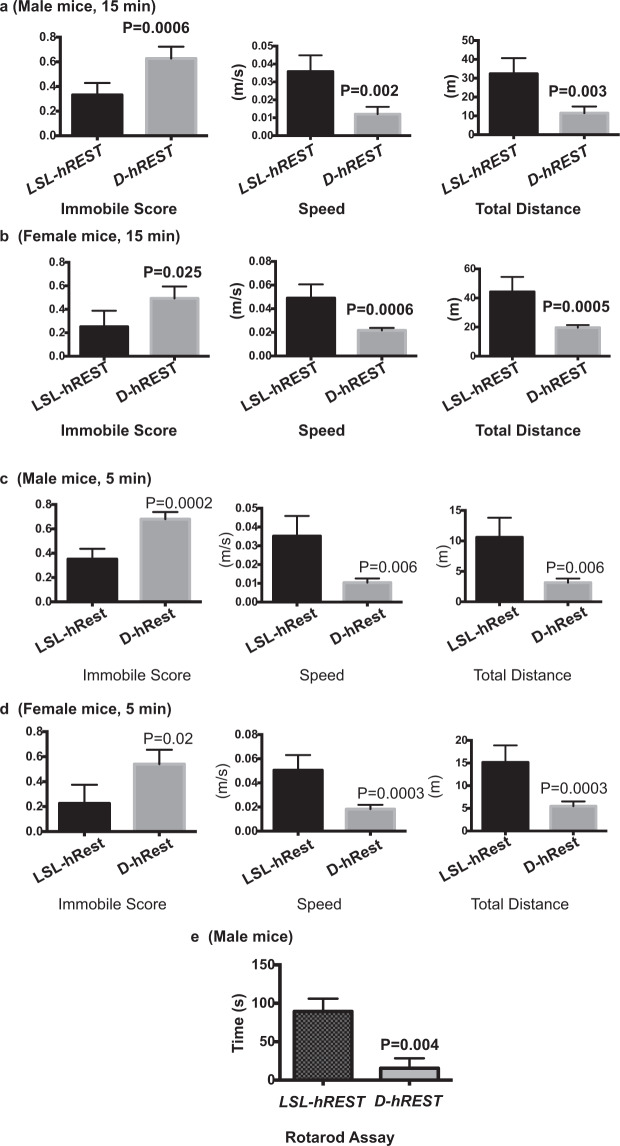
REST-DRD2 pathway regulates spontaneous locomotion in D-hREST mice. (**a**) *D-hREST* male mice showed significantly decreased spontaneous locomoter activity, as determined by open field assay performed for 15 min. Open-field data were calculated in terms of immobile score, speed, and total distance. P values are shown in the figure. N for *D-hREST* mice = 5; N for *LSL-hREST* mice = 5. (**b**) *D-hREST* female mice also showed significantly decreased spontaneous locomoter activity, as determined by open field assay performed for 15 min. P values are shown in the figure. N for *D-hREST* mice = 3; N for *LSL-hREST* mice = 7. **(c,d)** Both *D-hREST* male (**c**) and female (**d**) mice showed significantly decreased spontaneous locomoter activity when assayed for 5 min. The experiments were performed using the open field assay and the data were calculated in terms of immobile score, speed, and total distance. P values are shown in the figure. N for *D-hREST* male mice = 5; N for *LSL-hREST* male mice = 5. N for *D-hREST* female mice = 3; N for *LSL-hREST* female mice = 7. (**e**) Motor coordination test using the rotarod assay in male mice. N for each group = 8. P values are shown in the figure.

We took 10- to 12-week-old male mice, subjected them to spontaneous open field locomotion tests for 15 minutes, and analyzed the data using the ANY-maze video-tracking system and software. As compared to their *LSL-hREST* littermates, the *D-hREST* mice were significantly more immobile (hypokinesia) and slower (bradykinesia), which caused them to travel shorter distances (Fig. 7a). To determine whether the locomotion deficits seen here were dependent on sex, we performed the same tests in 10- to 12-week-old female mice. As shown (Fig. 7b), the locomotion deficits in the female mice were similar to those in their male counterparts. To determine whether the results were dependent on the time interval used for the assay, we repeated these experiments with separate groups of male and female mice for 5 minutes. The results were similar to what we observed for the 15-minute interval (Fig. 7c,d).

Because we did not see significant locomotion differences between male and female mice, we used only male mice in the rotarod assay to further test motor coordination. The results showed a robust locomotion deficit in *D-hREST* mice compared to their *LSL-hREST* littermates (Fig. 7e). Thus, both the spontaneous locomotion and the rotarod results phenocopied the locomotion deficits seen upon dopamine depletion, global DRD2 deletion, or specific DRD2 deletion from indirect-pathway medium spiny neurons (iMSNs)^43,47,48,50^.

### *D-hREST* and control littermate mouse brains had similar histological features and no apoptosis

**Figure 8:**
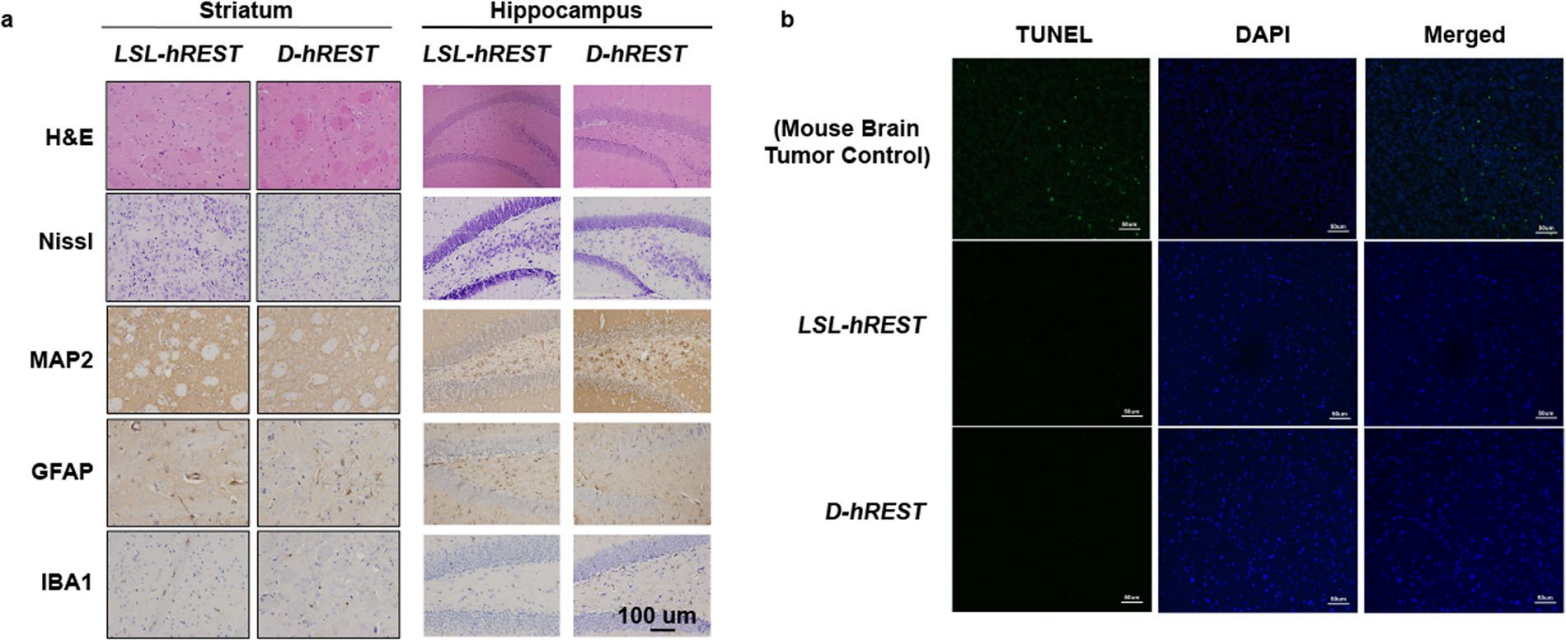
*D-hREST* and control littermate mouse brains had similar histological features and showed no apoptosis. (**a**) Striatum and hippocampus sections were stained with hematoxylin and eosin and cresyl violet (Nissl). Sections were also stained with anti-MAP2, anti-GFAP, and anti-IBA1. Scale bar = 100 μ. (**b**) Striatal sections were subjected to TUNEL assays. Mouse brain tumor sections were used as a positive control for the assay. In all experiments, age-matched *LSL-hREST* littermates were used as controls. Scale bar = 50 μ. Figures in both a and b represent at least three different images.

To determine whether the spontaneous locomotion deficits seen in the *D-hREST* mice were due to abnormal apoptosis caused by REST overexpression, we performed H&E and cresyl violet staining of mouse brain sections to assess the striatum and hippocampus in *D-hREST* mice and *LSL-hREST* control littermates for neuronal inclusions (Lewy bodies, pale bodies, neurofibrillary tangles, and other inclusions) or conspicuous neuronal loss (Fig. 8a). No neuronal inclusions or neuronal loss was identified in either group. On cresyl violet and MAP-2 immunostains, no abnormalities in cortical organization or neuronal morphology were identified in either group. No cavitary lesions indicative of vascular brain injury or white matter tract degeneration was identified. Neither group of animals exhibited acute or chronic inflammatory infiltrates in brain parenchyma. Regional heterogeneity in reactive astrogliosis and microglial activation were present and were similar in the *D-hREST* and *LSL-hREST* control littermates. An unblinded review of brain sections following the assessment described above did not reveal additional morphologic findings that could be used to confidently distinguish between *D-hREST* and *LSL-hREST* control littermate mouse brains. In addition, TUNEL assay exhibited no detectable apoptosis in the brain sections (striatum), although it exhibited a strong signal in a mouse brain tumor positive control (Fig. 8b). Thus, any effect of REST overexpression in *D-hREST* mice appears to not be attributable simply to morphological differences or apoptosis of REST- or DRD2-expressing cells.

## Discussion

Our work described here has produced a new mouse model of *hREST* overexpression. A study using full-length *Rest* knock-out mice indicated that REST is a neurogenesis repressor^16^. In that study, homozygous deletion of Rest in Nestin-expressing cells was embryonically lethal, as was hREST overexpression in our mice. The embryonic lethality of either complete deletion or overexpression of REST suggests that the amount of REST expressed is critical during embryonic neurogenesis.

Our results using these mice also suggest that REST represses DRD2 and that REST negatively regulates spontaneous locomotion *in vivo* when it is overexpressed in DRD2-expressing cells. These results are similar to the locomotion deficits seen in dopamine depletion or global DRD2 deletion. It is expected, but as yet unproven, that environmental salience would ameliorate the spontaneous locomotion deficit to some extent, as was seen in dopamine depletion models as well as in models of specific deletion of DRD2 in iMSNs^43,47,48,50^.

DRD2 is expressed in many types of neurons, even within the striatum, and, until recently, it was unclear which type(s) of neurons are involved in locomotion deficits. A recent mouse model has suggested that specific deletion of DRD2 from iMSNs triggers lateral inhibition of direct-pathway MSN activity, even in the presence of an intact dopamine transmission (as opposed to D2 autoreceptor deletion)^48^. These results suggest the critical, specific roles of iMSN and DRD2 in this process. We are currently examining the spatio-temporal specificity of REST-DRD2 axis–mediated locomotion deficits by expressing REST in iMSNs, cholinergic interneurons, and dopamine neurons. Because REST likely functions in a developmental stage-specific manner, we are also examining whether REST regulates locomotion function differently in adolescent versus adult mice. It is interesting that we did not see any impact of sex on the locomotion deficits, at least in terms of the immobile score, speed, and total distance. While males exhibit greater locomotion deficits in some diseases such as PD, this is less pronounced in human HD. Thus, our mouse model parallels human HD in this respect.

HD produces cognitive and behavioral disorders in addition to motor deficits^26,28,43,47,48,50^. Various rodent models have been created to study HD^51^. Although these models have produced a wealth of knowledge, none of them completely recapitulates the human disease, presumably because of the inherent structural and anatomical differences between the human and rodent brains and the short life span of rodents. Although, at this point, it is unclear whether our mouse model represents any of the movement disorders, it begins to shed light on the motor deficits seen in HD. Whether these mice also exhibit the other aspects of HD is unclear at this point and requires further investigation. It is unlikely that overexpression of REST alone would explain all the deficits produced in human HD. It is interesting that the motor deficits produced in our mouse model occur without major histological changes or differences in cell death; this suggests that a change in the dopamine circuitry alone–without major cell death–is sufficient to affect spontaneous locomotion. Indeed, some HD patients have been found to have no significant cell loss^52^. Thus, our mouse model provides an experimental system to study the dopamine circuitry in relation to motor deficits in the future.

HD is likely caused by a widespread alteration in biological and neural networks caused by mutant *HTT*. Interestingly, our results exhibiting a global alteration in the transcriptome profiles of *N-hREST* mouse brains suggest that REST can potentially regulate thousands of genes. Our *D-hREST* is a promising mouse model for further study of how various pathways might contribute to the locomotion deficits seen here or studies of other potential disorders.

REST functions in a context-dependent manner. The functional impact of REST overexpression in DRD2-expressing cells is likely to be different from that in other cell types. Many neurodegenerative diseases, such as Alzheimer’s disease (AD), PD, and HD, have some overlapping symptoms, particularly in terms of cognitive dysfunction. However, whereas neurodegeneration in AD is mainly seen in the cortical areas of the brain, in PD and HD it involves subcortical areas: dopaminergic neurons of the substantia nigra in PD and medium spiny neurons of the neocortex in HD. In that sense, HD and PD might have more mechanisms in common with each other than with AD. Thus, the context-dependent functions of REST might explain why our results described here suggest a harmful effect of REST overexpression, similar to those observed in HD^30–32,35^ and potentially in PD^38,39,53^, while another study has found a protective effect of REST overexpression in AD^18^.

At present there is no efficient mechanism-based therapy for HD^29^. Our results suggest that REST inhibitors would ameliorate the locomotion deficits seen in the current study and could be useful in HD treatment. Indeed, inhibition of REST overexpression in HD experimental models has been attempted using various approaches such as antisense oligonucleotides^35^ and small-molecule inhibitors^36^. However, at present, there is no robust REST-specific inhibitor that can be used *in vivo*. We previously created a recombinant form of REST, REST-VP16, by replacing the two-repressor domain of REST with the strong activation domain VP16^54^. REST-VP16 binds to the same DNA binding site as REST and serves as an activator instead of a repressor of REST targets. We have found that expression of REST-VP16 in neural stem/progenitor cells was sufficient to activate REST target genes and convert them into functional neurons^55^. We have also found that REST-VP16-mediated activation of REST target genes in committed myoblasts was sufficient to convert the myoblasts into a physiologically active neuronal phenotype^56^. In addition, the myoblast-derived neuronal cells, when transplanted into the cerebellum of P3 postnatal mice, a site of extensive postnatal neurogenesis, form glutamatergic neurons and generate synaptic connections with the endogenous neurons^57^. Thus, REST-VP16 appears to efficiently and specifically inhibit REST-mediated functions. We are currently examining whether adeno-associated virus-mediated delivery of REST-VP16 can ameliorate REST-mediated spontaneous locomotion deficits in *D-hREST*^*ov*^^/+^ mice.

## Methods

### Generation of REST conditional overexpression mouse model

The *LSL-hREST* mouse line was designed by SM and genOway and was established by genOway. Targeting constructs were prepared by assembling the *CAGGS-loxP-Neo/STOP-loxP-hREST/IRES/Luciferase-hGH polyA* sequence into an expression vector and then introducing the construct into the Rosa26 locus through electroporation into C57BL/6 embryonic stem cells (genOway). Targeted clones were identified by Southern hybridization with external probes. The strategy for construction of the targeting vectors is detailed in Fig. 1. Germline transmission was achieved by mating high-percentage chimeric males to wild-type females. *LSL-hREST* homozygous mice were identified through polymerase chain reaction (PCR) genotyping and then subsequently bred to *nestin-cre* (Jackson Laboratory) and *drd2-cre* mice (ER44; MMRRC). For the tracking of REST expression in the heterozygous *Drd2-cre;hREST* (*D-hRESTov*/+ = *D-hREST*) mice, they were further crossed with yellow fluorescent protein (YFP)-tagged reporter mice^58^. All mice were housed in a conventional facility with a 12-hour light/dark schedule. All procedures were approved by The University of Texas MD Anderson Cancer Center Animal Care and Use Committee and all methods were performed in accordance with the relevant guidelines and regulations.

### Immunofluorescence assays

These experiments were performed as described previously^21,23,54–57,59^. Mice were anesthetized and perfused with phosphate-buffered saline followed by 4% paraformaldehyde (PFA). Brain tissues were then dissected and fixed in 4% PFA overnight at 4 °C. Fixed brain tissues were processed for paraffin embedding and then cut into 5-μm sections. Methods used for immunofluorescent staining have been previously described^60^. Primary antibodies used for immunofluorescent staining were anti-REST (HPA006079, Sigma), anti-Drd2 (sc-5303, Santa Cruz Biotechnology), anti-Sox2 (ab171380, Abcam), and anti-Nestin (ab6142, Abcam). TUNEL assay was performed using a TUNEL apoptosis kit purchased from Roche (11684817910). Secondary antibodies used in immunofluorescent staining were conjugated with either Alexa 488 or Alexa 555 (Life Science Technologies). Staining results were viewed and photographed using confocal microscopy.

### RNA preparation and quantitative PCR (qPCR) for *hREST* and *Drd2* transcripts

These experiments were performed as previously described^21,23,54–57,59^. Total mRNAs were extracted, using TRIzol reagents (Invitrogen) according to the manufacturer’s instructions, from three sources: forebrains of E18.5 *N- hRESTov*/+ mice and their *LSL-hREST* littermates, striata of 2-month-old *D-hRESTov*/+ mice and their *LSL-hREST* littermates, and YFP-sorted brain cells of the *D-hRESTov*/+ mice and their *LSL-hREST* littermates. Approximately 1 μg total RNA was used as a template for cDNA synthesis using the Verso cDNA kit (Thermo Fisher Scientific). Quantitative real-time PCR ( qRT-PCR) was done using SYBR green master-mix (Applied Biosystems) as per the manufacturer’s instructions. All qRT-PCR experiments were done on an ABI7900HT sequence detection system (Applied Biosystems). The primer sets for detecting *hREST* and *Drd2* were *TCACAATGGGCCTAAACCTC/CGTGGGTTCACATGTAGCTCT* and *TTGTTCTTGGTGTGTTCATC/TATAGATGATGGGGTTCACG*, respectively.

### Transcriptome analyses using RNA sequencing

These experiments were performed as we have described previously^23,59^. To determine unbiased transcriptome profiles of *LSL-hREST* and *N-hREST*, we analyzed the RNA sequencing (RNA-Seq) data using Agilent GeneSpring GX11.5 software. To determine whether the subtypes corresponded to different functional groups, we performed a gene set enrichment analysis (GSEA) using the GSEA tool developed by the Broad Institute (www.broadinstitute.org/gsea). GSEA uses a collection of differentially expressed gene sets, annotated with the gene ontology biological processes, from the Molecular Signatures Database. We performed a further clustering analysis using the top 20 gene sets from each subtype and the robust R program language (www.r-project.org). We then identified the top 20 gene sets expressed in *LSL-hREST* versus *N-hREST* samples. The top gene sets in *LSL-hREST* were dominated by pathways involved in cell surface receptor signaling, postsynaptic membrane potential, chromatin assembly, DNA packaging, synaptic signaling, and negative regulation of gene expression. In contrast, the top gene sets in the *N-hREST* brains were dominated by pathways involved in immune response.

### Chromatin immunoprecipitation assay

ChIP assays of cultured neurospheres obtained from E12.5 wild-type mouse brains were performed as described previously^21,23^. Cells were fixed with 1% formaldehyde for 10 min in an incubator (37 **°**C, 5% CO_2_). After being washed with phosphate-buffered saline, the cells were immediately lysed with RIPA lysis buffer (supplemented with proteinase and phosphatase inhibitors) and subjected to sonication. The power of the sonication was adjusted to achieve a chromatin size of ~500 base pairs. After the debris was removed through centrifugation, REST antibody (#07–579, Millipore) or control rabbit IgG antibody was added to the supernatant and incubated at 4 **°**C overnight. REST-binding chromatin was purified by using Protein-G magnetic beads (#53033, Active Motif). Reverse cross-linking was done by incubating the samples at 65 **°**C for 4 hours. The resulting DNA strands were subsequently analyzed for specific regions on the *Drd2* promoter by using qRT-PCR. The primers used for the assays were as follows: Site #1, Forward primer: GCCCTATGGCTTGAAGGTAA, Reverse primer: GACTCAGTGGAAAGGGTGCT; Site #2, Forward primer: TCCACAACCATGCTTTCCAC, Reverse primer: GCACACAGGTTCAAGATGCT. Site #1 is extremely GC-rich, making it practically impossible to design primer sets capable of providing a robust PCR signal even when using the direct input of DNA prior to immunoprecipitation. The primer set used is 134 bp outside the REST binding site but within the 500 bp sheared DNA fragment size. The signal obtained from this primer set is therefore likely to represent underestimation of REST binding.

### Locomotion assays

These experiments were performed as we have described previously^47^,^61^. All behavioral assays were carried out on 10 to 12 weeks old, age-matched, *D-hREST* and *LSL-hREST* control mice. Each mouse was individually housed and trained according to the treatment it would receive.

### Open field assay

Open field testing was performed as previously described (Kelly *et al*., 1998; Dobbs *et al*., 2016). Briefly, an individual male mouse was placed in a 40 × 40-cm open-field arena. The movement of the mouse was recorded by a USB webcam connected to video capture software (ANY-maze) for 5 minutes. The recorded video files were analyzed using the ANY-maze software for total distance traveled, travel speed, and immobile score. The open field arena was cleaned with 70% ethanol between each trial.

### Rotarod assay

Mice were trained on the rotarod apparatus for at least 5 minutes three times each day for 3 days prior to the test. On the test day, two to four male mice were placed on a horizontally oriented, rotating cylinder (rod) suspended above a cage floor. The mice naturally try to stay on the rotating rotarod and avoid falling to the ground. The length of time that each mouse stayed on the rotating rod was measured by a digital panel controlled by the metal pad where the mouse landed.

### Histology and cresyl violet staining of D-hREST and LSL-hREST mouse brains

Representative blocks were prepared of *LSL-hREST* and *D-hRESTov*/+ mouse brains, including the cortex, subcortical nuclei, olfactory bulb, base of brain, brainstem, and cerebellum. The protocol for neuropathologic examination included examination of hematoxylin and eosin (H&E)– and cresyl violet–stained sections, as well as immunostains for microglia (IBA1, Wako, 019–19741, rabbit polyclonal, 1:250), neurons (MAP-2, 17490-1-AP, Proteintech, rabbit polyclonal, 1:100), and astrocytes (GFAP, ab7260, Abcam, rabbit polyclonal, 1:1000). Cresyl violet (Nissl) staining was performed on paraffin-embedded tissue sectioned at 10 µm, mounted on charged slides, and dried overnight at 60 °C. Following the deparaffinization and rehydration steps, slides were stained in a 1% cresyl violet solution (100 ml with 0.25 ml glacial acetic acid) for 1 hour at 60 °C. Slides were washed and developed in 96% alcohol, cleared in several changes of xylene, and mounted (Permount, Thermo Fisher Scientific).

### Immunostaining of D-hREST and LSL-hREST mouse brains

These experiments were performed as we have described previously^21,23,56,57,59^. Immunostaining was performed on paraffin-embedded tissue sectioned at 5 µm, mounted on charged slides, and dried overnight at 60 °C. Sections were deparaffinized and rehydrated. Heat-based antigen retrieval was performed using a 1× antigen retrieval solution at pH 9 (Agilent Technologies) carried out for 1 hour (30 minutes at 95 °C followed by 30 minutes on ice). All washing steps were carried out using a commercial Tris-buffered saline solution (1×) containing Tween 20, pH 7.6 (Agilent Technologies), and a 3% hydrogen peroxide solution (VWR International) was used to block endogenous peroxidase. Primary antibody was applied overnight at 4 °C following a 1-hour blocking step at room temperature with 2.5% horse serum (Vector Laboratories). Slides were thoroughly washed and the ImmPress horseradish peroxidase anti-rabbit IgG detection kit (Vector Laboratories) was applied for 1 hour at room temperature. Following additional washing steps, the target antigen was visualized using DAB chromogen in substrate buffer (Agilent Technologies), H&E counterstaining was applied, and the slides were rinsed in xylene and mounted with Permount.

### Neuropathologic analysis

H&E–, cresyl violet–, and immuno-stained wild-type and REST mouse brain sections were reviewed by a neuropathologist (MDCC) blinded to the mutation status. H&E– and cresyl violet–stained sections were used to assess the tissues for neuronal inclusions (Lewy bodies, pale bodies, neurofibrillary tangles, and other inclusions) or conspicuous neuronal loss. Neuronal morphology was examined on H&E–, cresyl violet–, and MAP-2–stained sections. Reactive astrogliosis, as an additional marker of tissue injury, was assessed by H&E– and GFAP–stained sections. Microglial activation was assessed in H&E– and IBA1–stained sections.

### TUNEL assays

TUNEL assays were performed according to the manufacturer’s instructions (Roche) as we have described previously^23^. Briefly, after hydrating the paraffin sections through alcohol grades, antigen retrieval was performed and the slides were blocked for 1 hour in 3% bovine serum albumin and 20% fetal bovine serum. The slides were then washed and incubated with TUNEL reaction mixture at 37 °C in a humidified atmosphere, followed by mounting with DAPI. Positive cells were counted in 10 different fields.

### Experimental design and statistical analyses

All statistical analyses were calculated using PRISM6 software (GraphPad). Significant differences between the *D-hREST* and *LSL-hREST* groups were analyzed using Student’s t-test. A P value of <0.05 was considered statistically significant. The results of statistical significance tests are described in each figure. All quantified data represent at least three independent experiments. All image data represent at least three independent areas. Microarray data were analyzed with Agilent GeneSpring GX11.5 and Ingenuity Pathway Analysis software.

### Data availability

The datasets generated and/or analyzed during the current study are available from the corresponding author on reasonable request.

## Electronic supplementary material

Supplementary Information
