# Author Correction: REST overexpression in mice causes deficits in spontaneous locomotion

**DOI:** 10.1038/s41598-022-25607-2

**Published:** 2022-12-16

**Authors:** Li Lu, Anantha Marisetty, Bin Liu, Mohamed Mostafa Kamal, Joy Gumin, Bethany Veo, YouQing Cai, Dina Hamada Kassem, Connie Weng, Mark E. Maynard, Kimberly N. Hood, Gregory N. Fuller, Zhizhong Z. Pan, Matthew D. Cykowski, Pramod K. Dash, Sadhan Majumder

**Affiliations:** 1 Departments of Genetics, The University of Texas MD Anderson Cancer Center, Houston, TX 77030 USA; 2 Department of Neurosurgery, The University of Texas MD Anderson Cancer Center, Houston, TX 77030 USA; 3 Department of Pain Medicine, The University of Texas MD Anderson Cancer Center, Houston, TX 77030 USA; 4 Department of Neurobiology and Anatomy, The University of Texas McGovern Medical School, Houston, TX 77030 USA; 5 Department of Pathology, The University of Texas MD Anderson Cancer Center, Houston, TX 77030 USA; 6 Department of Pathology and Genomic Medicine, Houston Methodist Hospital, Houston, TX 77030 USA; 7 Department of Neuro-Oncology, The University of Texas MD Anderson Cancer Center, Houston, TX 77030 USA; 8 Present Address: Baylor College of Medicine, Houston, TX 77030 USA; 9 Present Address: Department of Neurosurgery, MD Anderson Cancer Center, Houston, TX 77030 USA; 10 Present Address: Department of Epigenetics and Molecular Carcinogenesis, MD Anderson Cancer Center, Houston, TX 77030 USA; 11 Present Address: Department of Biochemistry, Faculty of Pharmacy, Ain Shams University, Cairo, Egypt; 12 Present Address: Department of Pediatrics/Hematology and Oncology, University of Colorado Denver Anschutz Medical Campus, Aurora, CO 80045 USA

Correction to: *Scientific Reports* 10.1038/s41598-018-29441-3, published online 14 August 2018

This article contains an error in the Data Availability statement.

The datasets generated and/or analyzed during the current study are available from the corresponding author on reasonable request.

should read:

The datasets generated and/or analyzed during the current study are available from the corresponding author on reasonable request. RNA-seq data is available from GEO with the accession number: GSE214743

