# Supplementary material for: REST overexpression in mice causes deficits in spontaneous locomotion
Source: Sci Rep. 2018 Aug 14;8:12083. doi: 10.1038/s41598-018-29441-3 (PMC6092433; doi:10.1038/s41598-018-29441-3)
Supplement: Supplementary file 1 — Supplementary Information [file 41598_2018_29441_MOESM1_ESM.pdf]

Supplementary materials

REST overexpression in mice causes deficits in spontaneous locomotion

Li Lu[1], Anantha Marisetty[1], Bin Liu[1], Mohamed Mostafa Kamal[1], Joy Gumin[2], Bethany Veo[1], YouQing Cai[3], Dina Hamada Kassem[1], Connie Weng[1], Mark Maynard[4], Kimberly N. Hood[4], Gregory N. Fuller[5], Zhizhong Z. Pan[3], Matthew D. Cykowski[6], Pramod Dash[4], and Sadhan Majumder[1,7,*]

Departments of [1]Genetics, [2]Neurosurgery, [3]Pain Medicine, [5]Pathology, and [7]Neuro-oncology, The University of Texas MD Anderson Cancer Center, Houston, TX 77030; [4]Department of Neuroscience, The University of Texas Medical School, Houston, TX 77030; [6]Methodist Hospital, Houston, TX 77030

Supplementary Table S1. Gene enrichment in *LSL-hREST* mouse brain

Gene Enrichment in LSL-hREST			
Gene Sets	SIZE	ES	FDR q-val
GO_CELL_SURFACE_RECEPTOR_SIGNALING_PATHWAY_INVOLVED_IN_CELL_CELL_SIGNALING	17	-0.73	0
GO_REGULATION_OF_POSTSYNAPTIC_MEMBRANE_POTENTIAL	16	-0.71	0.001
GO_CHROMATIN_ASSEMBLY_OR_DISASSEMBLY	28	-0.6	0.002
GO_DNA_PACKAGING	34	-0.57	0.003
GO_DNA_CONFORMATION_CHANGE	37	-0.55	0.003
GO_PROTEIN_SECRETION	17	-0.67	0.004
GO_SYNAPTIC_SIGNALING	89	-0.46	0.004
GO_CELL_CELL_SIGNALING	116	-0.44	0.004
GO_NEGATIVE_REGULATION_OF_GENE_EXPRESSION_EPIGENETIC	21	-0.62	0.004
GO_REGULATION_OF_MEMBRANE_POTENTIAL	59	-0.48	0.004
GO_CHROMATIN_SILENCING	21	-0.62	0.006
GO_MODULATION_OF_SYNAPTIC_TRANSMISSION	60	-0.47	0.011
GO_POSITIVE_REGULATION_OF_SYNAPTIC_TRANSMISSION	20	-0.6	0.011
GO_INORGANIC_ION_TRANSMEMBRANE_TRANSPORT	68	-0.45	0.012
GO_DNA_REPLICATION_DEPENDENT_NUCLEOSOME_ORGANIZATION	15	-0.65	0.013
GO_PROTEIN_DNA_COMPLEX_SUBUNIT_ORGANIZATION	31	-0.54	0.013
GO_PROTEIN_HETEROTETRAMERIZATION	17	-0.6	0.014
GO_AMIDE_TRANSPORT	15	-0.63	0.013
GO_PEPTIDE_TRANSPORT	15	-0.63	0.013
GO_ION_TRANSMEMBRANE_TRANSPORT	91	-0.42	0.013

(Size: Number of genes in the gene sets after filtering out the genes not in the expression dataset (RNA-seq); ES: Enrichment score for the gene set; FDR q-Val: False discovery rate; GO: Gene Ontology)

Supplementary Table S2. Gene enrichment in *N-hREST* mouse brain

Gene Enrichment in *N-hREST* Group

Gene Sets	SIZE	ES	FDR q-val
GO_POSITIVE_REGULATION_OF_IMMUNE_RESPONSE	42	0.54	0
GO_INNATE_IMMUNE_RESPONSE	45	0.53	0
GO_ACTIVATION_OF_IMMUNE_RESPONSE	39	0.55	0
GO_PATTERN_RECOGNITION_RECEPTOR_SIGNALING_PATHWAY	16	0.73	0
GO_POSITIVE_REGULATION_OF_INNATE_IMMUNE_RESPONSE	23	0.61	0
GO_ACTIVATION_OF_INNATE_IMMUNE_RESPONSE	22	0.61	0
GO_LEUKOCYTE_ACTIVATION	43	0.48	0
GO_POSITIVE_REGULATION_OF_IMMUNE_SYSTEM_PROCESS	64	0.45	0
GO_REGULATION_OF_IMMUNE_RESPONSE	63	0.43	0
GO_LEUKOCYTE_DIFFERENTIATION	31	0.53	0
GO_POSITIVE_REGULATION_OF_DEFENSE_RESPONSE	30	0.53	0
GO_IMMUNE_RESPONSE	72	0.41	0
GO_IMMUNE_EFFECTOR_PROCESS	41	0.46	0
GO_LYMPHOCYTE_DIFFERENTIATION	22	0.55	0
GO_TUBE_MORPHOGENESIS	41	0.44	0
GO_CELL_ACTIVATION	54	0.42	0
GO_CELLULAR_RESPONSE_TO_BIOTIC_STIMULUS	15	0.64	0.001
GO_TUBE_DEVELOPMENT	54	0.42	0.001
GO_DEFENSE_RESPONSE	87	0.36	0.001
GO_POSITIVE_REGULATION_OF_DNA_METABOLIC_PROCESS	20	0.57	0.001

(Size: Number of genes in the gene sets after filtering out the genes not in the expression dataset (RNA-seq); ES: Enrichment score for the gene set; FDR q-Val: False discovery rate; GO: Gene Ontology)
